# Supplementary material for: A pcyt-1 allelic series reveals in vivo consequences of reduced phosphatidylcholine synthesis in C. elegans
Source: G3 (Bethesda). 2026 Jul 4;16(8):jkag161. doi: 10.1093/g3journal/jkag161 (PMC13439949; doi:10.1093/g3journal/jkag161)
Supplement: Supplementary file 1 [file jkag161_supplementary_data.zip › Supplementary_Material_G3-2026-406823.pdf]

SUPPLEMENTARY MATERIAL

Suppl. Fig. S1. Lipidomics reveals a hierarchy of *pcyt-1* allele. (A) and **(B)** Show the fatty acid composition of phosphatidylcholines and phosphatidylethanolamines of L4 worms growth at 25°C and of the indicated genotypes; this data is included in the heat map from Fig. 6. For this analysis, the mol% fatty acid composition of phosphatidylcholines and phosphatidylethanolamines was determined using UPLC-MS/MS. For each fatty acid, the mean mol% among the samples was determined and set to 0 (zero) and the variance adjusted to 1, thus giving equal weight to all fatty acids. Note that the 15:0, 17:0 and 19:0 species likely consist mostly of mmBCFAs while the 17:1 and 19:1 species are likely mostly the dietary cyclopropanes 17:0 delta and 19:0 delta, respectively. **(C)** Volcano plot of PC and PE species in *pcyt-1(C211Y)* vs control N2 worms at 20°C.

Suppl. Movies 1-4
Z-stack of PCYT-1::mNeonGreen (WT for Movies 1 and 3; P154A variant for Movies 2 and 4) grown for 24 hours at either 20°C (Movies 1-2) or 25°C (Movies 3-4) post L1 stage.

Suppl. Fig. S1

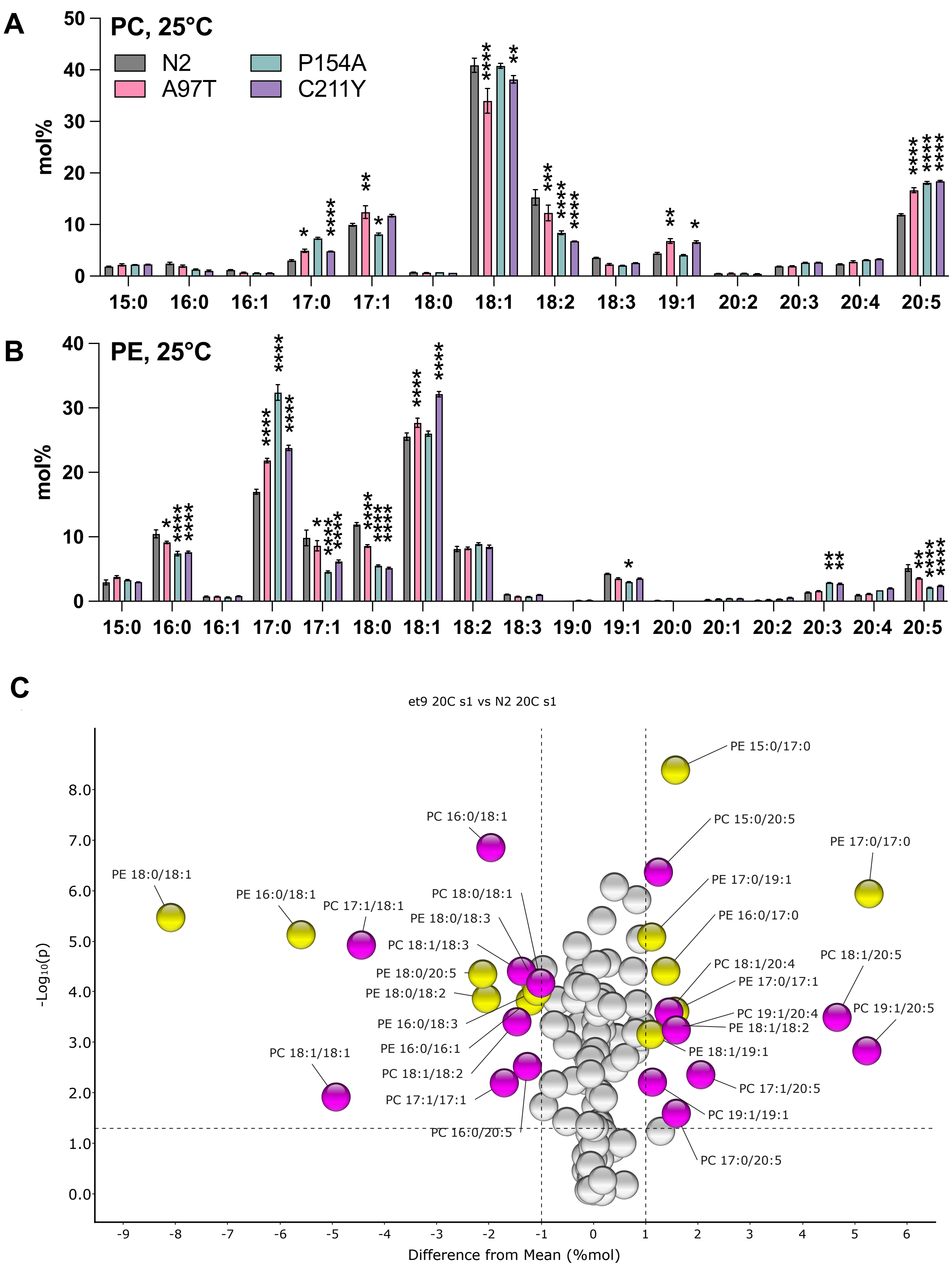

A
PC, 25°C
N2
A97T
P154A
C211Y
mol%
15:0 16:0 16:1 17:0 17:1 18:0 18:1 18:2 18:3 19:1 20:2 20:3 20:4 20:5
B
PE, 25°C
mol%
15:0 16:0 16:1 17:0 17:1 18:0 18:1 18:2 18:3 19:0 19:1 20:0 20:1 20:2 20:3 20:4 20:5
C
et9 20C s1 vs N2 20C s1
-Log10(p)
Difference from Mean (%mol)
PE 15:0/17:0
PC 16:0/18:1
PC 15:0/20:5
PE 17:0/17:0
PE 18:0/18:1
PE 16:0/18:1
PC 17:1/18:1
PC 18:0/18:1
PE 18:0/18:3
PE 17:0/19:1
PE 16:0/17:0
PC 18:1/18:3
PE 18:0/20:5
PE 18:0/18:2
PC 18:1/20:4
PE 17:0/17:1
PC 18:1/20:5
PE 16:0/18:3
PC 19:1/20:4
PE 18:1/18:2
PC 19:1/20:5
PE 16:0/16:1
PE 18:1/19:1
PC 18:1/18:1
PC 18:1/18:2
PC 17:1/20:5
PC 17:1/17:1
PC 19:1/19:1
PC 16:0/20:5
PC 17:0/20:5
